# An in vitro lung model to assess true shunt fraction by multiple inert gas elimination

**DOI:** 10.1371/journal.pone.0184212

**Published:** 2017-09-06

**Authors:** Balamurugan Varadarajan, Andreas Vogt, Volker Hartwich, Rakesh Vasireddy, Jolanda Consiglio, Beate Hugi-Mayr, Balthasar Eberle

**Affiliations:** 1 Department of Anesthesiology and Pain Medicine, Inselspital, Bern University Hospital, University of Bern, Bern, Switzerland; 2 Department of Cardiac and Vascular Surgery, Inselspital, Bern University Hospital, University of Bern, Bern, Switzerland; Medical Center of the Johannes Gutenberg-University, GERMANY

## Abstract

The Multiple Inert Gas Elimination Technique, based on Micropore Membrane Inlet Mass Spectrometry, (MMIMS-MIGET) has been designed as a rapid and direct method to assess the full range of ventilation-to-perfusion (V/Q) ratios. MMIMS-MIGET distributions have not been assessed in an experimental setup with predefined V/Q-distributions. We aimed (I) to construct a novel in vitro lung model (IVLM) for the simulation of predefined V/Q distributions with five gas exchange compartments and (II) to correlate shunt fractions derived from MMIMS-MIGET with preset reference shunt values of the IVLM. Five hollow-fiber membrane oxygenators switched in parallel within a closed extracorporeal oxygenation circuit were ventilated with sweep gas (V) and perfused with human red cell suspension or saline (Q). Inert gas solution was infused into the perfusion circuit of the gas exchange assembly. Sweep gas flow (V) was kept constant and reference shunt fractions (IVLM-S) were established by bypassing one or more oxygenators with perfusate flow (Q). The derived shunt fractions (MM-S) were determined using MIGET by MMIMS from the retention data. Shunt derived by MMIMS-MIGET correlated well with preset reference shunt fractions. The in vitro lung model is a convenient system for the setup of predefined true shunt fractions in validation of MMIMS-MIGET.

## Introduction

The worldwide volume of major surgical procedures is estimated at 243 million per year [1]. Morbidity and mortality after major surgery are associated particularly with postoperative pulmonary complications like lung injury and acute respiratory distress syndrome (ARDS) [2, 3].

A worldwide multicenter observational study yielded a prevalence of ARDS due to all causes of 10.5% among ICUs in 50 countries [4]. The Berlin definition of ARDS introduced a mild (200mmHg < PaO2/FIO2 ≤ 300mmHg), moderate (100mmHg < PaO2/FIO2 ≤ 200mmHg) and severe oxygenation stage (PaO2/FIO2 ≤ 100mmHg) of the syndrome [5]. ARDS hospital mortality of mild stage was 34.9%, of moderate stage 40.3% and of severe stage 46.1% [4, 6]. However, there was evidence of under recognition and under treatment of this globally public health problem indicating a potential for improvement of health care for this patient cohort [4]. Shunt and CT scan based mean lung weight increased with severity of stage [5]. In a retrospective observational ARDS study systematic thoracic CT scans yielded most commonly consolidations (94.1%) and ground glass opacities (85.3%) and health care was changed in 26.5% of cases due to the results obtained from CT scans[7]. The use of extracorporeal membrane oxygenation (ECMO), providing gas exchange without the need for mechanical ventilation in ARDS, increased by over 400% between 2006 and 2012 in the United States [8].

Hypoxemia due to insufficient pulmonary gas exchange is a common reason for ICU care of patients. However, information from readily available parameters like arterial PO_2_, arterial PO_2_/FIO_2_ ratio, physiological shunt and deadspace is quite limited and error-prone due to misinterpretation. Since P.D. Wagner’s studies in the early 1970’s, the multiple inert gas elimination technique (MIGET) has provided useful insights into the physiology and pathophysiology of gas exchange and hypoxemia, making it the reference method for the assessment of ventilation-to-perfusion matching [9]. In the 2000’s a novel MIGET variant was introduced that analyzed inert gas by micropore membrane inlet mass spectrometry (MMIMS) instead of gas chromatography [10]. Compared to the conventional GC-MIGET, MMIMS-MIGET appears to offer substantial advancement through reduction of analysis time, sample volume, material and human resources [11]. MMIMS-MIGET shunt (MM-S) calculated from a single-pore MMIMS has been shown to correlate well with Riley shunt in a porcine lavage lung model [12]. An additional improvement in the current MMIMS-MIGET setup was the use of a multi-pore [11] probe instead of the single-pore probe used in previous studies [10, 12]. The multi-pore variant offers an approximately 400-fold increased sensitivity in its inert gas partial pressure measurement, resulting in much less susceptibility for technical and analytical errors.

The conventional MIGET, as well as its underlying concept, have withstood the test of time in numerous in vivo studies and was currently used in a volunteer study to evaluate a novel functional proton magnetic resonance imaging technique to measure regional V_*A*_/Q ratio in the lung [13]. MIGET and its MMIMS variant have also been compared with each other in animal experiments by Kretzschmar et al [11]. MIGET by MMIMS was feasible to evaluate the impact of pressure support ventilation in a porcine sepsis lung injury model [14] or to assess V_*A*_/Q distributions during cardiopulmonary resuscitation in anaesthetized pigs [15]. In summary MIGET has been proven valuable to get deeper insight to gas exchange and V_*A*_/Q distributions in various animal and clinical experiments. So far, however, the use of a bench model to test the validity, accuracy and precision, and reproducibility of these techniques has not been described.

Currently, as part of modern artificial organ support systems, blood-gas exchangers or oxygenators are regularly used in clinical practice to replace (cardiopulmonary bypass) or support the gas exchange function of the lung (ECMO and/or CO2 removal) [16]. Studies involving such hollow fiber membrane oxygenators have reported a good correlation between measured oxygen and carbon dioxide transfer with model-predicted gas exchange [17]. In a similar experimental setup as in [14], Borland and colleagues presented a membrane oxygenator as a model for lung nitric oxide and carbon monoxide transfer [18].

The general aim of this study is to test the accuracy, precision and reproducibility of the MMIMS variant of MIGET shunt fractions under the stable and adjustable in vitro conditions of an extracorporeal perfusion and membrane oxygenation circuit. Fitted with several parallel “lung” compartments governing gas exchange, the in vitro lung model (IVLM) appears to provide a simple and robust model for creating predefined V/Q relationships. We hypothesize that in an appropriate IVLM setup, various reference shunt fractions can be defined using controlled sweep gas and perfusion flow, which then allows comparison with shunt fractions determined using MMIMS-MIGET.

The specific aims of this study were (I) to design an IVLM with five separate gas exchange compartments connected in parallel to achieve resolution of shunt fractions between 0.0 and 1.0, and (II) to compare shunt fractions derived from MMIMS-MIGET with preset reference shunt fractions of the IVLM (IVLM-S). We also aimed to compare measurements utilizing saline as a priming and perfusion fluid with those using a human red cell suspension.

## Materials and methods

### Experimental setup of IVLM

**Fig 1:**
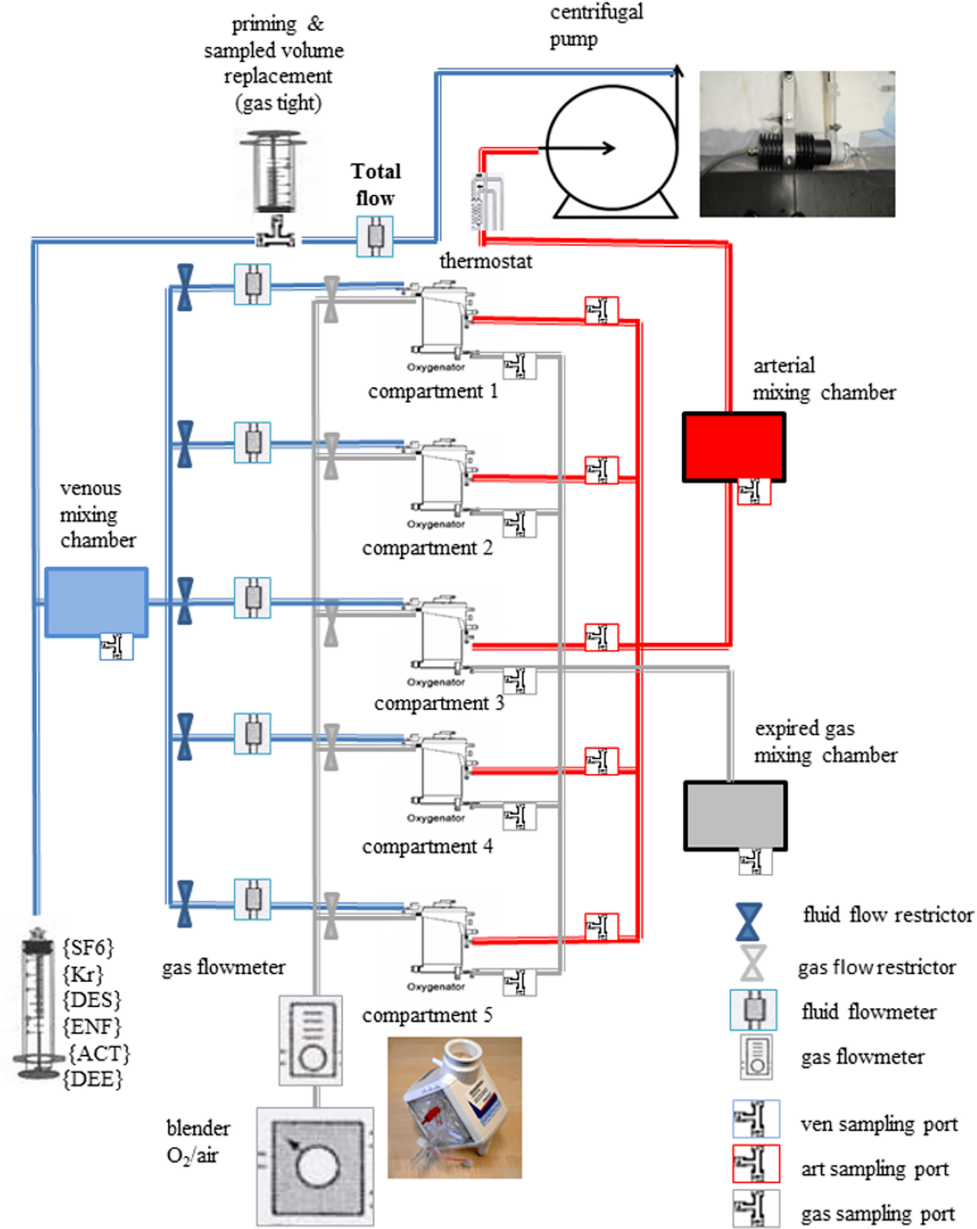
Schematic of in vitro lung model setup.

The perfusion and gas exchange circuit of the IVLM consists of the following components assembled as shown in Fig 1:

1. Pump: A micro-diagonal pump (DeltaStream DP-II, Medos,Stolberg/D) generating non-pulsatile perfusate flow, at a maximum rate of 2500 ml/min within the present setup.
2. Gas exchangers: Five pediatric membrane gas exchange units (QUADROX-i Pediatric Oxygenators; MAQUET, Hirrlingen, Germany) in a parallel assembly. These hollow-fiber microporous membrane oxygenators each contain two chambers; the first consists of gas-permeable polypropylene fiber mats alternating with heat exchange mats made of polyurethane, while the second chamber contains only gas-conducting polypropylene fiber mats arranged at 90° to each other in order to improve perfusate mixing outside the fibers and hence, gas exchange. (www.maquet.com) Polypropylene fibers are permeable to anesthetic gases through the micro pores. Each of these oxygenators could be perfused at up to 2500 ml/min perfusate flow and ventilated with sweep gas (air-oxygen mixture) using a gas blender (Maquet Cardiopulmonary AG, Hirrlingen, Germany).
  Total and compartmental perfusate flows were measured using in-line liquid flow meters (Levitronix, Zurich, Switzerland). Total gas flow to the oxygenators was measured using a thermal mass flow meter (TSI, Shoreview, MN, USA).
3. Fluorinated ethylene propylene circuit tubing (Tygon®, Saint-Gobain performance plastics, Akron, OH, USA) which minimizes loss of inert gas from perfusate.
4. Priming fluid was either human RBC (SRBC) suspended in fresh frozen plasma and saline at a hematocrit of 40 to 45% or normal saline (SNS)– 0.9% NaCl. Priming volume was 2500 ml.
5. Heat exchanger: Warm water (38°C) was circulated through all oxygenator units using an external heater/cooler system with temperature control (HCV, Type 20–602, JostraFumedica, Muri/CH). A thermistor (Sams, 3M Health Care, Ann Arbor, MI, USA) was connected to its fluid reservoir outlet for temperature monitoring.

The system was designed to be devoid of leaks, and the inlet ports were connected to the bottom of the mixing chambers in order to facilitate air bubble free priming. In order to avoid entrainment of air bubbles, the system was vented, pre-filled with priming fluid, and any remaining air bubbles were removed prior to initiating an experiment.

### Circuit prime

Human blood and saline were used as priming fluids. In the first experiment, expired human blood products were used to simulate in vivo conditions using a target hematocrit of 40 to 45%. This was produced by mixing 500 ml packed red blood cells with 500 ml fresh frozen plasma and 1500 ml normal saline. The blood products were obtained from the blood bank of the University Hospital of Bern and identity of the donors was not available. Hence the source of these blood products was completely de-identified. In the second experiment, the priming solution utilized was 2500 ml of normal saline.

### Perfusate flow measurement

Liquid flow meters (Levitronix, Zürich, Switzerland) measured perfusate flow in the model. These flow meters function based on ultrasonic flow measurement principle with a measurement error of ± 1% of the true value and the reproducibility error is less than ± 0.5% of the true value. The difference in transit times of the piezo-electric pulses propagated along and against flow is a measure of average fluid velocity along the path of ultrasonic beam. Total perfusate flow was measured with a main flow meter, while flow distribution to the five oxygenators was quantified by individual, in-line perfusate flow meters.

### Sweep gas flow measurement

The model was ventilated with air as sweep gas using a gas blender (Maquet Cardiopulmonary AG, Hirrlingen, Germany). Individual gas flows to the oxygenators were estimated using a thermal mass flow meter (TSI, Shoreview, MN, USA).

### Perfusate mixing chambers

Two cylindrical mixing chambers made of special glass (Trabold&CoAG, Bern, CH) are located in the common inlet upstream, as well as in the common outlet downstream of the gas exchanger assembly. The inlet chamber representing the “venous side” has a single input port receiving perfusate from the pump, and five outlet ports connected to each gas exchange unit, which are fitted with sampling ports. Inert gas solution was introduced upstream to this mixing chamber via a three-way stopcock. The outlet (“arterial”) mixing chamber downstream of the gas exchanger assembly has five inlet ports receiving perfusate from each of the gas exchangers, and a single outlet port fitted with a sampling port. Perfusate then returns to the pump (Fig 1). Thus, the upstream mixing chamber of the IVLM represents the mixed venous compartment of pulmonary blood volume while the downstream represents the pulmonary venous compartment or, in the presence of introduced anatomic shunt, also the systemic arterial compartment. The circulating perfusate together with added inert gas solution was gently mixed due to the surface design inside the mixing chambers. The outlet from the downstream mixing chamber is connected to the inlet line of the centrifugal pump.

### Preset shunt flow measurement

The scenario of a normal lung with V/Q ratio approximating 1 was simulated in the IVLM by maintaining equal sweep gas (V) and fluid (Q) flows. In a single-unit gas exchange circuit both V and Q were set to 2500 ml/min, whereas 500 ml/min per gas exchange unit was maintained in a five-oxygenator circuit.

Intrapulmonary shunt occurs when functional lung units are perfused in a non-aerated state, i.e., when their regional V/Q ratio is zero. To achieve this state in the model, one or more gas exchangers, and hence sweep gas contact, were bypassed by shunt tubes with defined flow (IVLM shunt, IVLM-S).

Reference shunt fraction was calculated as:

presetfractionalshunt=measuredshuntedflow/measuredtotalflowtoupstreammixingchamber

For a modeled shunt fraction of 0.2, one of the five gas exchangers were bypassed, thus eliminating its perfusate flow. Its respective shunt tube flow was manually adjusted to 500 ml/min using the individual upstream perfusate flow valves. All resultant flows were verified using the in-line flow meters. Analogous maneuvers allowed shunting 2, 3, or 4 gas exchange units, with respective shunt fractions then measured and calculated.

Selection of preset shunt fractions was conducted in a randomized manner, in order to prevent systematic effects of residual disequilibrium shortly after step changes in inert gas concentrations. After changing inert gas infusion rate or shunt fraction, an equilibration period of no less than 15 min was allowed prior to MMIMS-MIGET sampling.

### Preparation and administration of inert gas solution for MMIMS-MIGET

The inert gas infusate [10] was prepared in a gas-tight 500 ml normal saline bag (500 ml) by equilibrating gaseous sulfur hexafluoride (SF6, 90 ml gas) and krypton (Kr, 24 ml gas) with the fluid, followed by the addition of liquid desflurane (DES, 100 μl), enflurane (ENF, 100 μl), diethyl ether (DEE, 100 μl), and acetone (AC, 1 ml). Inert gas dose and resultant concentrations were selected according to the sensitivity of the MMIMS device determined during previous IVLM experimentation. Inert gas solution was infused into the circuit upstream to the inlet mixing chamber, using a volumetric infusion pump (Alaris GP, Cardinal Health, 1180 Rolle, Switzerland) at a rate representing approximatly1/1000th of the total pump flow. To maintain a meticulously bubble-free and closed system, two air-tight glass syringes remained attached to the inlet port of the upstream glass mixing chamber in order to allow for limited volume changes within the circuit (e.g., from inert gas infusion, sampling).

### MMIMS MIGET with Multipore Probe

The theory and practice of multiple inert gas analysis and the derivation of ventilation-perfusion distributions using the novel MMIMS-based variant of the MIGET have been described extensively; in this study they were performed accordingly [10–12]. The version of MMIMS technology used in this study utilizes a multi-pore MMIMS probe with 200 pores. Additionally, current software updates (Version: MMIMSMainV43, Oscillogy LLC, USA) were performed in order to enhance sensitivity and signal strength, to shorten measurement time, and to facilitate data analysis. When compared to previous studies with single-pore MMIMS or conventional MIGET using time-consuming gas chromatography, multi-pore MMIMS-MIGET demonstrated considerably improved sample turnover time and hence, better temporal resolution (sampling intervals of 15 min are feasible, with 8 min for each sample analysis).

### Measurement of inert gas concentrations and MMIMS-MIGET-derived shunt fraction

After ruling out fluid leaks or air bubbles, the centrifugal pump was started at 2500 ml/min. Temperature was controlled by a heat exchanger set to 38°. After a few minutes’ equilibration time, inert gas infusion was started and maintained for at least 15 min to achieve steady-state inert gas concentrations. For each selected reference shunt setting, duplicate samples were drawn at 15-minute intervals using gas-tight glass syringes (Cadence Science Inc®, Plainfield Pike Cranston, RI) spiked with an anticoagulant (heparin).

With 0, 1, 2, 3 or 4 out of 5 oxygenators bypassed to achieve predefined reference shunts, bubble-free samples were collected simultaneously from up- and downstream mixing chambers and immediately processed for inert gas analysis using the MMIMS system (Beta Version 1.0, Oscillogy®, Folsom, PA, USA). Retentions were computed from the ratio of downstream partial pressure to upstream partial pressure for each inert gas [12]. Although a similar sampling approach could be employed to acquire mixed expired samples in order to compute excretion, this proved to be impractical due to the inherent difficulty in handling highly soluble gases (DEE & Acetone) with the present setup. Retention data and solubility coefficients were converted, according to MIGET methodology as described by Evans and Wagner[19], into a distribution of perfusion (as percentage of total perfusate flow) over 50 compartments with defined ventilation-to-perfusion ratios. Fractional MMIMS-MIGET shunt in the compartment of interest was defined as MMS ≡V/Q < 0.005.

### Statistical analysis

Using Wagner’s algorithm [20] programmed on Labview 8.0 (National Instruments, Austin, TX), MMIMS-MIGET shunt fractions were calculated from inert gas concentrations. All metric data are expressed as mean ± SD. Correlation between predefined shunt from IVLM and MMIMS-MIGET shunt was described by linear regression and agreement with Bland-Altman analysis [21, 22]. Origin 8.0 was used for data management and analysis (linear regression, Bland-Altman analysis). As used in previous studies, the degree of experimental error was inferred from the residual sum of squares (RSS) [12, 23]. Data analysis was carried out in an explorative manner, with p < 0.05 considered as significant.

## Results

The IVLM allowed stable control of compartmental perfusate and sweep gas flows, as well as reproducible inert gas transfer. Duplicate samples were taken at each reference shunt setting; hence 10 samples were taken at five different time points. The reported IVLM shunt values were calculated from the measured ventilation and perfusion flowrates during the experiment in order to avoid the influence of handling errors.

Fractional shunt measurements were compared for asanguineous perfusate (n = 10), ranging from 0 to 0.79 (IVLM-SNS) and from 0 to 0.81 (MM-SNS), respectively. For red blood cell containing perfusate, IVLM-SRBC ranged from 0 to 0.91 and measured MM-SRBC from 0 to 0.83. As an indicator of experimental error, the MMIMS dataset had a residual sum of squares (RSS) < 5 in 75% and RSS < 10 in 95%.

### Correlation and agreement of IVLM-S and MM-S

**Fig 2:**
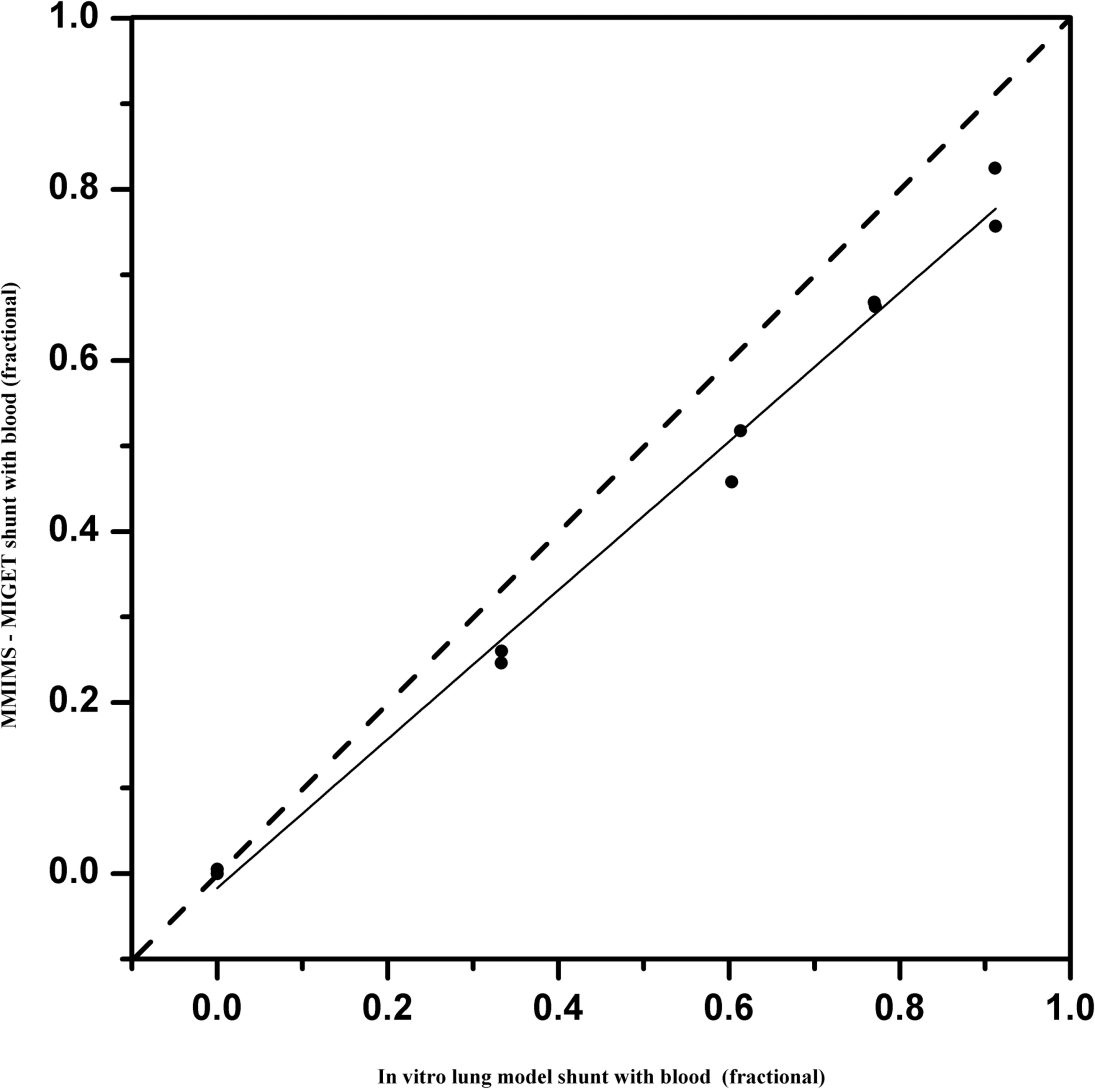
Linear regression analysis with blood as priming fluid. Linear regression analysis for MMIMS-MIGET based shunt fraction–with blood as priming fluid (MM-SRBC) on predefined in vitro lung model shunt (IVLM-SRBC): MM-SRBC = 0.87*IVLM-SRBC-0.02 (r2 = 0.96, P< 0.0001). Duplicate data from 0 to 0.8 model shunt fractions included. Solid line = linear regression; Dashed line = line of identity.

**Fig 3:**
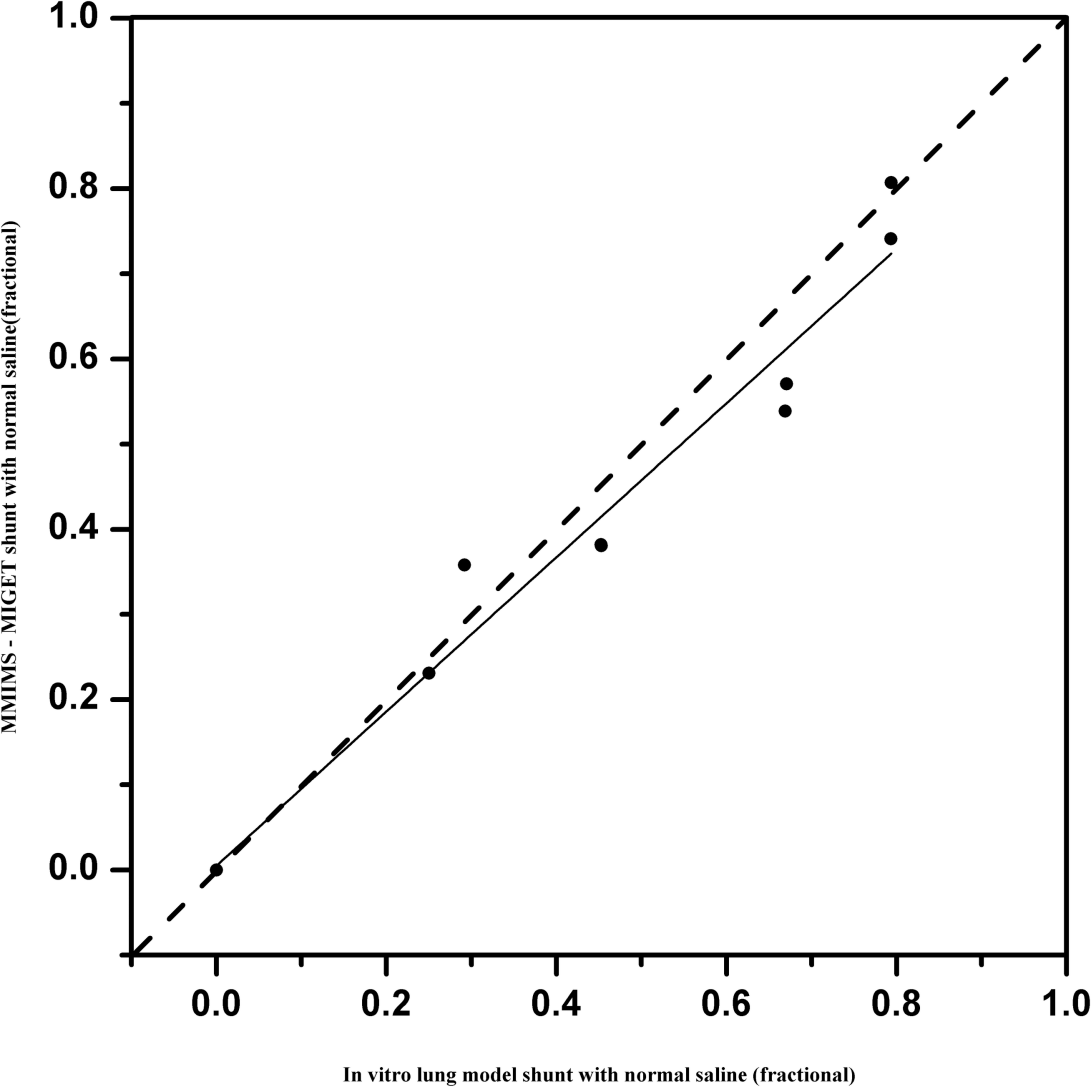
Linear regression analysis with saline as priming fluid. Linear regression analysis for MMIMS-MIGET shunt fraction–with saline as priming fluid (MM-SNS) on predefined in vitro lung model shunt (IVLM-SNS): MM-SNS = 0.91*IVLM-SNS +0.005 (r^2^ = 0.99, P< 0.0001). Duplicate data from 0 to 0.8 model shunt fractions included. Solid line = linear regression; Dashed line = line of identity.

Correlation between IVLM-S and MM-S was examined for 10 data pairs using linear regression analysis. As an example, Fig 2 illustrates the association between IVLM-SRBC vs MM-SRBC (r2 = 0.96; P < 0.0001). Fig 3 demonstrates the respective association for saline perfusate (IVLM-SNS vs MM-SNS, r2 = 0.99; P < 0.0001; duplicate sample data at each model shunt).

**Fig 4:**
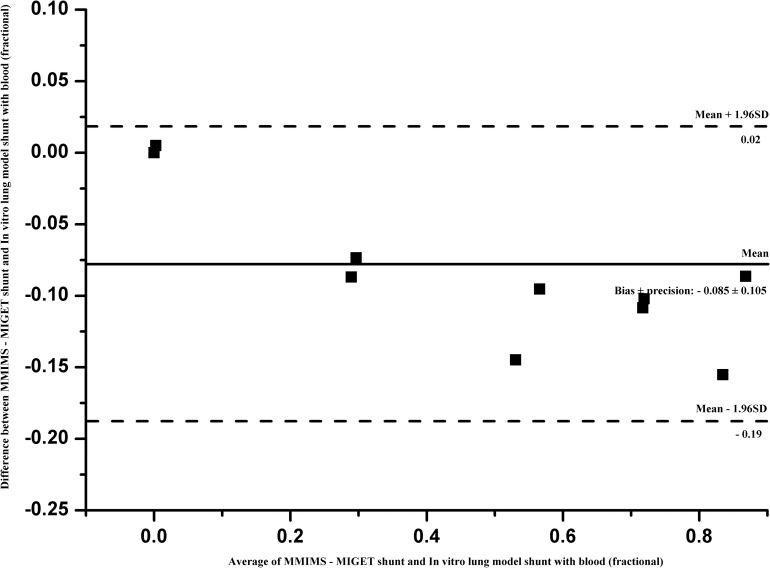
Bland-Altman analysis with blood as priming fluid. Bland-Altman analysis of MMIMS-MIGET based shunt fraction–with blood as priming fluid (MM-SRBC) on predefined in vitro lung model shunt (IVLM-SRBC). Bias ± precision (2 SD) was -0.085 ± 0.11 with 95% limits of agreement (dashed) of -0.19 and 0.02.

**Fig 5:**
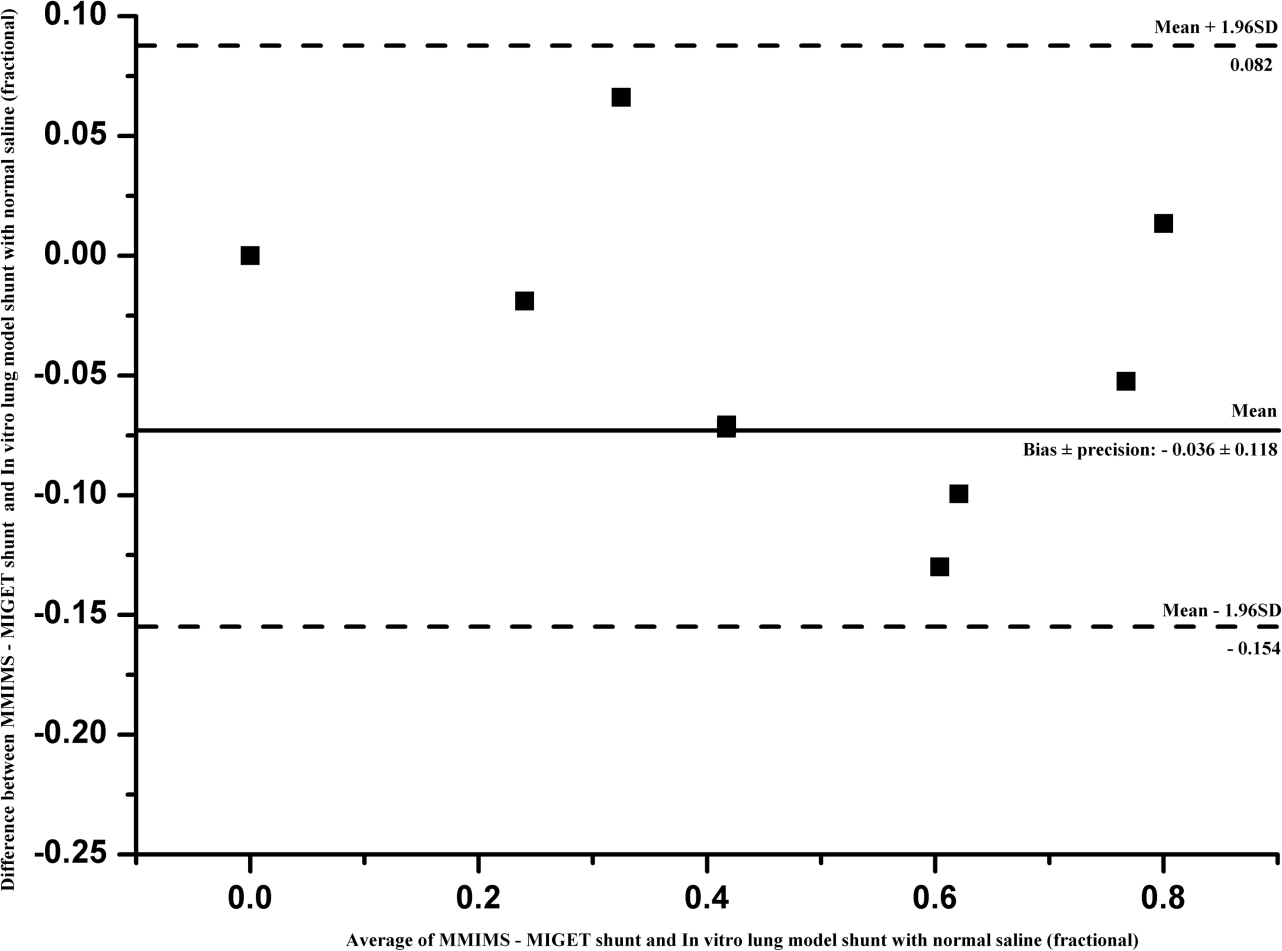
Bland-Altman analysis with saline as priming fluid. Bland-Altman analysis of MMIMS-MIGET based shunt fraction–with saline as priming fluid (MM-SNS) on predefined in vitro lung model shunt (IVLM-SNS). Bias ± precision (2 SD) was -0.04 00B1 0.12 with 95% limits of agreement (dashed) of -0.154 and 0.082.

MM-S underestimated IVLM-S both in blood (bias ± 2 SD = - 0.085 ± 0.105) and saline perfusate (bias ± 2 SD = - 0.036 ± 0.118). Bland-Altman plots of the difference between MM-S and IVLM-S are shown in Fig 4 (blood) and Fig 5 (saline). Overall coefficient of variation for MM-SRBC was 4.8% and 10.2% for MM-SNS.

## Discussion

This study demonstrates, as its primary finding, that the measurement of shunt fraction using MMIMS-MIGET technology is valid in determining true shunt fraction in an in-vitro lung model with artificial gas exchange. Good correlation exists between the measured MM-S and the preset IVLM-S over a wide range of shunt fractions. Our results indicate a 3–8.5% underestimation of true shunt by the MMIMS-MIGET based methodology for blood-containing, as well as asanguineous, perfusate (Figs 2 and 3). The overall precision was found to be good, with limits of agreement at ± 10% shunt fraction.

The purpose of constructing this in vitro model of compartmental pulmonary gas exchange was to provide a simple, robust and precise tool to produce predefined model V/Q relationships for comparison with V/Q relationships measured by MMIMS-MIGET. In many previous studies, MIGET was used as the “gold standard” for determination of true intrapulmonary shunt fraction [20, 24, 25]. By establishing physically predefined and controlled shunt fractions in the model, we are now able to utilize predefined IVLM shunt as the reference standard when evaluating MMIMS-MIGET shunt.

We found good correlation (MM-SRBC: r2–0.99, MM-SNS: r2–0.96) of the tested method to reference shunt; for clinical purposes, bias and precision appear satisfactory. However, the slight bias and imprecision may not be exclusively due to error in MMIMS-MIGET. Similar to any reference technique in this field, an IVLM does not categorically exclude potential sources of systematic and unsystematic error. For example, as with potential error within MMIMS-MIGET, the respective determination of shunt fraction in the IVLM relies on SF6 excretion and retention. At the current stage of model development, we utilized retention data only, which limited our ability to minimize measurement error. Nevertheless, we found that the proportional bias of 0.085 in blood and 0.036 in saline perfusate in the current study, using a multi-pore MMIMS version, was already substantially improved compared to 0.15 in a previous series from our group [12], in which single-pore MMIMS-MIGET-based shunt determination had been compared to Riley shunt measurement in an animal model of lung injury. This may also indicate that there are still various residual sources of systematic error in setups comparing MMIMS-MIGET shunt to reference techniques. The overall error may include measurement errors, sampling errors, and recovering shunt fractions from retention data [19]. Correlation of IVLM-S with MM-S yielded a bias comparable to previously published studies. The bias values of this study were similar to 0.04 obtained by Kretzschmar et.al, which assesses agreement between conventional MIGET with gas chromatography and MMIMS-MIGET [11]. In the animal study of Duenges et al., the range of possible shunt fractions was restricted due to the limitations of lavage injury, whereas the full range in our IVLM may improve numerical correlation.

In our current system, results from saline (IVLM-SNS Vs MM-SNS) and blood (IVLM-SRBC Vs MM-SRBC) have demonstrated satisfactory bias and precision. We thus conclude that crystalloid prime may be conveniently used for testing of reproducibility and further technical evolution of the IVLM setup; however, MMIMS-MIGET validation series in a clinically relevant range of V/Q ratios will also require priming with realistic hematocrit levels or even with whole blood.

The bias still indicated an underestimation of true shunt by MMIMS-MIGET in both blood and saline. A better automated lung model with automated perfusate and sweep gas flow control may considerably reduce measurement error due to manual handling of the IVLM. A limitation of this study is that precise settings of shunt in the IVLM, pertaining to the clinically relevant range, could not be achieved due to inherent difficulties in mechanical maneuvering of ventilation and perfusion flows. Upon automation of the lung model, we plan to perform more extensive experiments in the clinically relevant range of 0 to 0.4.

The true potential of the MMIMS-MIGET lies in the bedside measurements of the whole spectrum of ventilation-to-perfusion distributions and inequalities e.g. from shunt over a normal distribution to deadspace. In this study, we focused on introducing the in vitro lung model and its measurements of shunt in detail. Separate studies are being carried out with an automated lung model to realize other kinds of inequality scenarios in the model.

## Conclusion

Known true shunt fractions were generated in a novel, five-compartment in vitro model of gas exchange; using the multi-pore MMIMS-based MIGET system, we were able to measure shunt with satisfactory accuracy and precision. Our results suggest that such a model of compartmentalized gas exchange may represent a convenient system to validate and test MIGET systems and underlying assumptions against preset V/Q relationships. Translation of MMIMS-MIGET validation results from IVLM experimentation to animal and human studies will allow repetitive assessment of ventilation-perfusion distribution within time frames short enough to impact clinical decision making. Further refinements in the model, in terms of maneuverability and control, will enable the extension of the range of V/Q ratios that can be generated.

## Supporting information

S1 Data Data sets pertaining to MMIMS-MIGET samples. Data sets pertaining to MMIMS-MIGET derived IVLM shunt fractions with blood and saline as priming fluids.
(ZIP)
